# SND1 aggravates mitochondrial damage, apoptosis and extracellular matrix degradation in IL-1β-stimulated chondrocytes via PINK1/BECN1 pathway

**DOI:** 10.1186/s40001-023-01340-y

**Published:** 2023-09-25

**Authors:** Shufeng Lin, Huiyang Guo, Xiaoxuan You, Zefeng Zhang, Hui Ye

**Affiliations:** Department of Orthopedics, The Second Affiliated Hospital of Fujian Medical University, Quanzhou, 362000 Fujian China

**Keywords:** Osteoarthritis, SND1, Mitochondrial, Extracellular matrix, The PINK1, BECN1 pathway

## Abstract

Recently, evidence has suggested a regulatory role for SND1 in osteoarthritis progression. Interestingly, we found that SND1 protein expression was increased, mitochondria were shrunken and decreased in number, mitochondrial membrane potential was decreased, mitochondrial ROS production was increased, and ATP levels were decreased in IL-1β treated mouse chondrocytes, and SND1 silencing removed these changes. Furthermore, IL-1β treatment promoted inflammatory factor secretion in chondrocytes, promoted cell apoptosis, increased MMP13 protein and inhibited collagen II protein expression, and si-SND1 inhibited the IL-1β effects. We validated the association between SND1 and PINK1 and found that PINK1 reversed the inhibitory effects of SND1 silencing on IL-1β-induced mitochondrial damage, inflammatory reaction, apoptosis and extracellular matrix degradation in mouse chondrocytes. Furthermore, we found that PINK1 upregulated BECN1 protein expression and that BECN reversed the inhibitory effects of PINK1 silencing on IL-1β-induced mitochondrial damage, inflammatory reaction, apoptosis and extracellular matrix degradation. Further mechanistic studies revealed that PINK1 inhibited the AMPK/mTOR signaling axis to aggravate IL-1β induced mouse chondrocytes injury by upregulating BECN1 protein expression. In vivo results showed that the damage to cartilage tissue was significantly alleviated in rats with osteoarthritis by knocking down SND1 expression.

## Introduction

Osteoarthritis is a disease characterized by joint space narrowing, osteophyte formation, and cartilage degeneration [1]. Osteoarthritis is the most prevalent joint disease associated with aging, and in the context of increasing social aging, the rising incidence of osteoarthritis has become increasingly recognized as causing increasing harm, which can lead to loss of joint function and permanent disability and is currently the leading cause of dysfunction in the weight-bearing joints of the knee and hip [2]. Survey studies show that there are currently approximately 110 million patients with knee osteoarthritis in our country, and the prevalence of osteoarthritis is more than 30% among the elderly over 65 years old [3, 4].

The death of chondrocytes and the degeneration of cartilage are associated with multifaceted factors, which can be divided into chondrocyte intracellular factors and cartilage extracellular factors, of which intracellular factors account for the main aspects. As a key organelle in the cell, mitochondria are at the center of cellular metabolism and has important effect in the generation of ATP through oxidative phosphorylation [5]. In addition to providing energy to cells, mitochondria are the most important organelles for intracellular production of free radicals and regulation of cell death. It has been shown that mitochondrial functions, including mitochondrial respiratory chain activity and ATP synthesis, are altered in osteoarthritic chondrocytes [6–8]. Mitochondrial dysfunction may affect several specific pathways involved in pathology, including oxidative stress, chondrocyte apoptosis, chondrocyte inflammation with matrix catabolism, among others. Studies have shown that mitochondrial respiratory chain activity, mitochondrial membrane potential, and elimination of free radicals are reduced in osteoarthritic chondrocytes compared with normal chondrocytes.

Decreased oxidative respiratory chain activity in osteoarthritic chondrocytes leads to decreased ATP production, increased number of compensatory mitochondria, and generation of large amounts of ROS, which eventually cause mitochondrial dysfunction and accelerate chondrocyte death. Damaged mitochondria mostly appeared swollen morphologically, and the continuous fragmentation of the mitochondrial membrane was obvious, and the inner cristae structure was confused and blurred [9]. From a functional point of view, the elimination capacity of free radicals, the integrity of mitochondrial DNA, and the antioxidant capacity of these mitochondria were reduced. In addition, damaged mitochondria can also produce more cytochrome C, ROS and inflammatory factors, which further damage the normal physiological function of cells and promote apoptosis [10, 11].

The inducing factors of chondrocyte apoptosis mainly include various inflammatory factors, including IL-1β and TNF-α. IL-1β is the most prominent of all inflammatory mediators, which plays a leading role. In vitro osteoarthritis model, IL-1β-induced chondrocyte injury model is widely used. On one hand, IL-1β can significantly increase the degradation of extracellular matrix, on the other hand, it can also increase cartilage damage [12–14]. Therefore, the study of IL-1β induced chondrocyte apoptosis, cartilage extracellular matrix degradation and inflammatory response is of great significance for the treatment of osteoarthritis.

SND1 (staphylococcal nuclease and Tudor domain containing (1) protein, also known as Tudor-SN or p-100 protein, is evolutionarily highly conserved and widely expressed in various organisms including human, mouse, rat, cow, zebrafish and pea [15]. The human SND1 gene maps to position 7q31.3 and encodes a 910 amino acid, approximately 100 kDa protein consisting of four tandem repeats of staphylococcal nuclease like domains at the N-terminus and a TSN domain embedded with Tudor at the C-terminus [16]. Recently, it was shown that in chondrocytes and tissues of osteoarthritic rats, SND1 accelerated ferroptosis in cells by destabilizing HSPA5 mRNA and inhibiting HSPA5 expression to promote the degradation of GPX4, suggesting that SND1 may participate in oxidative stress in osteoarthritis chondrocytes [17]. However, the involvement of SND1 in the progression of osteoarthritis is poorly studied, and the specific regulatory mechanisms also require further investigation.

## Materials and methods

### Samples

Knee cartilage tissues from patients with osteoarthritis undergoing knee arthroplasty at our hospital were collected (*N* = 20, 61.3 ± 3.65 years), and non osteoarthritic cartilage tissue samples were obtained from fracture trauma patients (*N* = 20, 62.1 ± 3.24 years). All subjects signed informed consent for sample collection. The study protocol was approved by the Ethics Committee of the Second Affiliated Hospital of Fujian Medical University.

### Chondrocyte culture and transfection

Knee articular cartilage tissue of healthy male C57BL/6 mice (10 weeks; weighting 20–30 g) was collected. The specific operation is as follows: use ophthalmic scissors to separate the skin and muscle around the knee joint. The articular cartilage can be seen as transparent soft tissue with the naked eye. Use sharp forceps to passively separate the hyaline cartilage and place it in a Petri dish containing PBS. The soft tissue around hyaline cartilage was isolated, and then the cartilage tissue was placed in a sterile EP tube and cut. Add 1 ml of 0.25% trypsin to resuspend the cartilage tissue, put it into 37 °C incubator for digestion for 30 min, and shake it upside down every 5 min. The digestion was terminated after 30 min, and the pancreatic enzyme was discarded after centrifugation with 300 g in a centrifuge for 10 min. Then 3 ml of 1.5% collagenase type II was added, resuspended, transferred to a 15 ml centrifuge tube, and placed in a cell incubator for digestion for 6 h. The centrifuge tube was centrifuged at 300 g for 10 min in a low-speed centrifuge, resuspended using DMEM medium, and the cell suspension was transferred to a bottle for culture. When the cells proliferated to 90% confluence, they were digested with trypsin and passaged. The treatment of mouse chondrocyte inflammation model was as follows: add 1 μL/mL of IL-1β (PeproTech, USA) into 6-well plates (DMEM-F12 culture medium containing 10% fetal bovine serum and 1% double antibody in each well), 2 μL in each well, with a concentration of 10 ng/ml, and treat for 12 h. PBS with the same volume as IL-1β was added to mouse chondrocytes in another 6-well plate as a control group. The SND1 pcDNA3.1 vector (pcDNA-SND1), PINK1 pcDNA3.1 vector (pcDNA-PINK1), BECN1 pcDNA3.1 vector (pcDNA-BECN1) and vector were purchased from RiboBio Co., Ltd (Guangzhou, China) and transfected using RiboBio Transfection Kit. The SND1 siRNA, PINK1 siRNA and scramble were purchased from Santa Cruz Biotechnology (Santa Cruz, CA, USA).

### RT-qPCR

Cell Total RNA was isolated by the TRIzol. Single-stranded cDNA was synthesized with the PrimeScript Reagent Kit. Real-time qPCR was conducted using a SYBR Premix Ex TaqTM Kit. Sangon Biotech designed and synthesized the primers in the study. SND1 forward, 5ʹ-GTG ATC AGA TAC CGG CAG GAT G-3ʹ, reverse, 5ʹ-TCT TAA TAG CTC TGG CCT CTG CAG-3ʹ; GAPDH forward, 5ʹ-CAC CAT TGG CAA TGA GCG GTT C-3ʹ, reverse, 5ʹ-AGG TCT TTG CGG ATG TCC ACGT-3. The relative expression levels were normalized using the 2^−ΔΔCt^ method.

### Immunohistochemical assay

Cartilage tissue sections were digested in pancreatin for 10 min and then treated with 3% H_2_O_2_. Next, the cartilage tissue sections were blocked in 10% goat serum for 1 h, and then the sections were rinsed 2 times with PBS. The rinsed clean sections were incubated with anti-SND1 antibody (1:50) overnight at 4 °C in a refrigerator. Sections were rinsed 3 times with PBS and incubated with biotinylated secondary antibodies. Next, sections were rinsed and incubated with 3,30-diaminobenzidine (DAB) substrate for 2 min.

### ROS content detection

Cells (1 × 10^5^ density) were seeded in culture flasks, and after 24 h of culture, cells were incubated with 10 μM of DCFH-DA (MCE, New Jersey, USA) for 20 min in a 37 °C incubator. Next, cells were digested with pancreatin and a cell suspension was obtained. The cell suspension was centrifuged at 1,000 × g for 10 min, and rinsed twice with PBS, then the cells were obtained after centrifugation for flow cytometry detection. The DCFH-DA positive area was ROS fluorescence intensity.

### Flow cytometry

The cell culture medium was collected and rinsed twice with PBS. Next, the cell density (1 × 10^6^ cells/mL) was adjusted and 100 μL cell suspension was aspirated to inoculate into a cell culture flask, and then the cells were incubated with 5 μL of Annexin V-FITC (Sigma–Aldrich; USA) and propidium iodide in a dark environment. Finally, binding buffer was added in the culture tube, and flow cytometry was used to determine apoptosis rate.

### Western blotting

To detect the protein in the cartilage tissue of patients with osteoarthritis and the cartilage tissue of SD rats with osteoarthritis, chondrocytes should be isolated first, and then the protein in the cells should be extracted. Refer to “Chondrocyte culture and transfection” Section chondrocyte culture and transfection for the method. The protein in chondrocytes was extracted by RIPA lysis buffer (Beyotime Biotechnology, China). Then, proteins were transferred to PVDF membranes. After blocked for 2 h at 37 °C and incubated at primary antibodies overnight at 4 °C with primary antibodies (Abcam): GAPDH (1:3000), SND1 (1:2000), MMP13 (1:3000), collagen II (1:1500), PINK1 (1:1000), BECN1 (1:2000), AMPK (1:2000), mTOR (1:2500), p-AMPK (1:1000) and p-mTOR (1:1500). Then, the membranes were incubated with HRP-conjugated goat anti-rabbit IgG for 1 h at room temperature. The protein bands were visualized with ECL detection reagents and analyzed with ImageJ software (National Institutes of Health, Bethesda, MD, USA).

### Hematoxylin and eosin staining

Previously harvested tissue samples were fixed in 4% formalin overnight, and then the cartilage tissues were dehydrated by ethanol and embedded with paraffin and prepared into 5 μm-thick sections. The sections were next stained by hematoxylin and eosin and then observed under a light microscope (Leica Microsystems GmbH, Wetzlar, Germany).

### Co-IP assay

Cells were rinsed 2 times with PBS and then lysed extensively by adding RIPA lysis buffer. Next, the cells were collected and centrifuged to obtain the supernatant, which was then incubated with the corresponding antibodies overnight at 4 °C. Then, protein A agarose beads were added to obtain antigen antibody complexes and incubated overnight at 4 °C on a shaker. Next, the agarose bead antigen antibody complexes were collected by transient centrifugation and rinsed with PBS. Free antigen, antibody, and beads were obtained by adding protein loading buffer to the complexes and boiling. Finally, the expression levels of the obtained proteins were detected by Western blotting.

### MMP level detection

The cells were digested with pancreatin and the cell culture medium was collected, after which the cells were filtered through a screen mesh and subjected to cell counting (2 × 10^4^ cells). Next, 1 ml JC-1 working solution was added to resuspend the cells and incubated at 37 °C for 20 min in the dark. Cells were then centrifuged at 4 °C for 1500 rmp and rinsed with JC-1 staining buffer for 5 min. JC-1 staining buffer was finally added for resuspension of cells and detection of MMP content by flow cytometry.

### ATP content detection

Cells were trypsinized and media were collected, followed by addition of ATP lysate and ice bath for 10 min. Next, the lysates were centrifuged at 2000 × g centrifugation for 5 min to obtain the supernatant. 100 µL of ATP detection working solution (Beyotime Biotechnology; China) was added to a 96-well plate, standard diluent and 20 µL of sample were added after standing for 5 min at room temperature, then after thorough mixing, RUL was measured on a microplate reader and ATP concentration was calculated. The protein concentration was measured simultaneously, and the ratio of ATP concentration to protein concentration was taken as the result (nmoL/mg).

### Animals

Eighteen SPF grade male SD rats, 8–10 weeks old, weighing 280–300 g, were provided by the experimental animal center of Southern Medical University. The temperature of the feeding room is 22 ± 3 °C, the humidity is 55–60%, and 12 h alternates day and night. All rats were fed adaptively for 1 week before the follow-up test. SD rats were randomly divided into three groups with 6 rats in each group, namely sham group, osteoarthritis model group and si-SND1 group. The specific modeling procedure is as follows: all animals were weighed and skinned before surgery, and 10% chloral hydrate solution was intraperitoneally injected into anesthetized rats at a dose of 0.35–0.45 mL/kg. After that, a longitudinal incision was made at a position about 1 cm inside the right knee joint patella, and the dissection was layered until the knee joint cavity was exposed. The sham surgery group sutures each layer of tissue layer by layer without damaging any tissue; The modeling group dislocated the patella laterally, then flexed the knee joint to fully expose the anterior cruciate ligament and medial meniscus. Then, the medial meniscus was excised using ophthalmic scissors. Finally, the patella was repositioned, and the joint cavity was closed with physiological saline. The incision was sutured layer by layer. After the rats woke up, they returned to the cage for free movement. The first 3 days after surgery, penicillin was injected to prevent infection, and the padding was replaced every 3 days. On the 1th, 7th, 14st and 21st days after surgery, 100 µL of si-SND1 was injected into the knee joint cavity of rats in the treatment group; The modeling group was injected with an equal amount of negative control at the same time, and after 4 weeks, all groups of rats were euthanized and knee joint specimens were collected. The procedures for the care and use of animals have been approved by the Ethics Committee of the Second Affiliated Hospital of Fujian Medical University (2021-365).

### TUNEL staining

Cartilage tissue sections were fixed in paraformaldehyde for 30 min and then incubated with methanol solution for another 30 min. Subsequently, the sections were incubated with TUNEL reaction mixture for 1 h at room temperature, and then developed by DAP and observed under a microscope.

### Histological observation and scoring of rat knee joint

The Osteoarthritis Research Society International (OARSI) grading system was used to evaluate the pathological changes of osteoarthritis cartilage in the rat knee. 0 points represent no pathological changes of OA, and 24 points represent the most serious pathological changes of OA. Scores of 1–12 represent early pathological changes of OA. See references for specific scoring principles [18].

### Statistical analysis

The SPSS software was used to analyzed all dates (ver. 21.0). The measurement data are expressed in mean ± SD, which is consistent with the data of Normal distribution. The comparison between the two groups is conducted by t test. When comparing multiple groups, one-way ANOVA is used for homogeneity of variance, and nonparametric test is used for uneven variance; For non-Normal distribution, Mann–Whitney U test was used for comparison between two groups, and Kruskal–Wallis Test was used for comparison between multiple groups. *P* < 0.05 means statistically significant.

## Results

### SND1 expression is increased in cartilage tissue of osteoarthritis patients

**Fig. 1:**
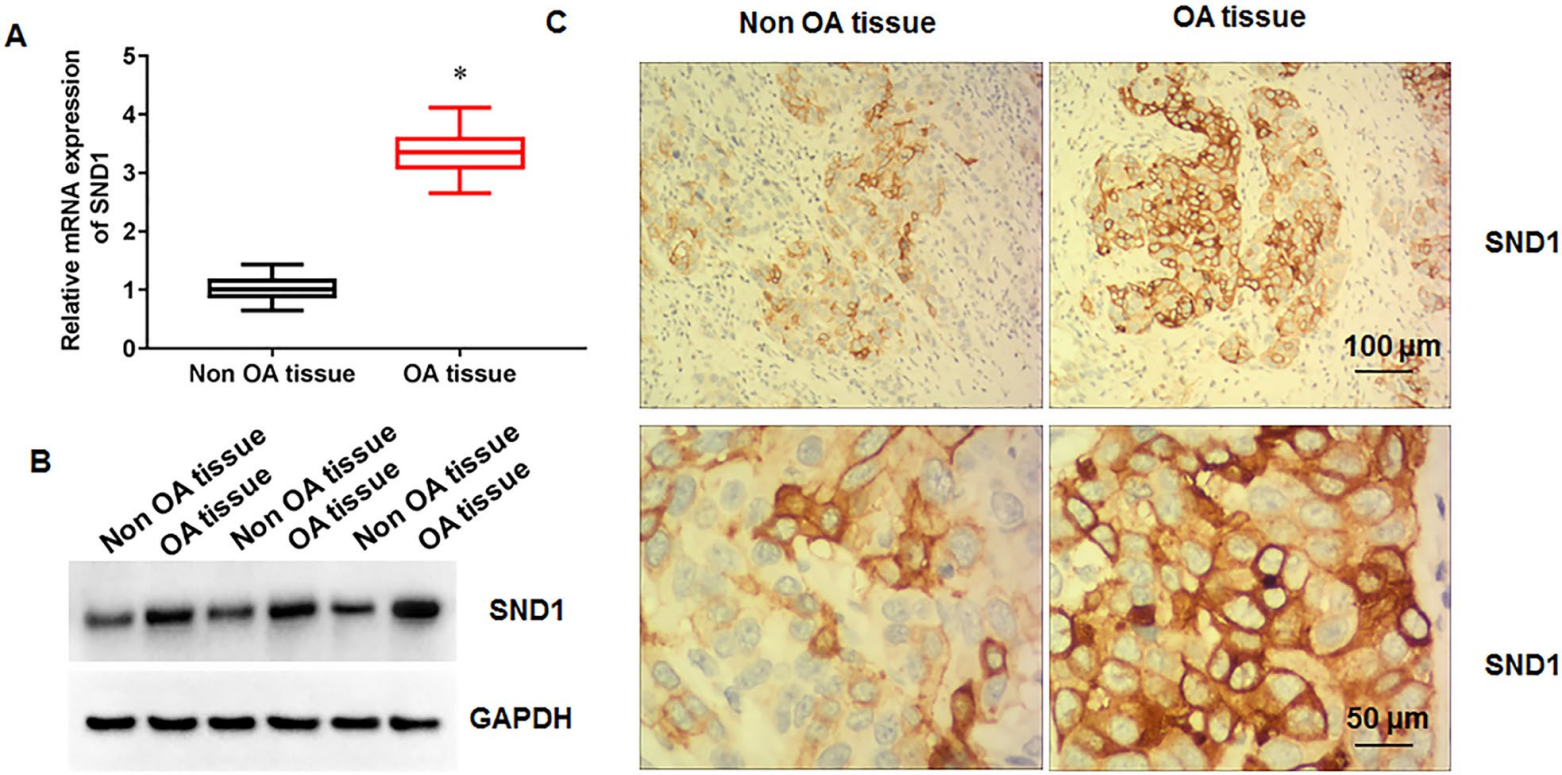
SND1 expression in cartilage tissues of osteoarthritis patients. **A**. SND1 mRNA expression in knee osteoarthritis cartilage samples (*N* = 20) and non-osteoarthritis cartilage samples (*N* = 20). **B**. SND1 protein expression in chondrocytes isolated from knee cartilage tissues from osteoarthritis patients and fracture trauma patients. **C**. Immunohistochemistry was used to detect SND1 expression. *N* = 6. *p < 0.01

We performed RT-qPCR analysis of knee osteoarthritis cartilage samples (*N* = 20) and non-osteoarthritis cartilage samples (*N* = 20) and showed that SND1 mRNA expression was upregulated in the cartilage of osteoarthritis patients (Fig. 1A). Moreover, the protein expression of SND1 was increased in chondrocytes isolated from knee cartilage tissue of patients with osteoarthritis (Fig. 1B). In addition, the immunohistochemical results suggested that SND1 positive cell numbers was increased in cartilage tissue of patients with osteoarthritis (Fig. 1C).

### Knockdown of SND1 alleviates mitochondrial damage and matrix degradation in IL-1β-treated chondrocytes

**Fig. 2:**
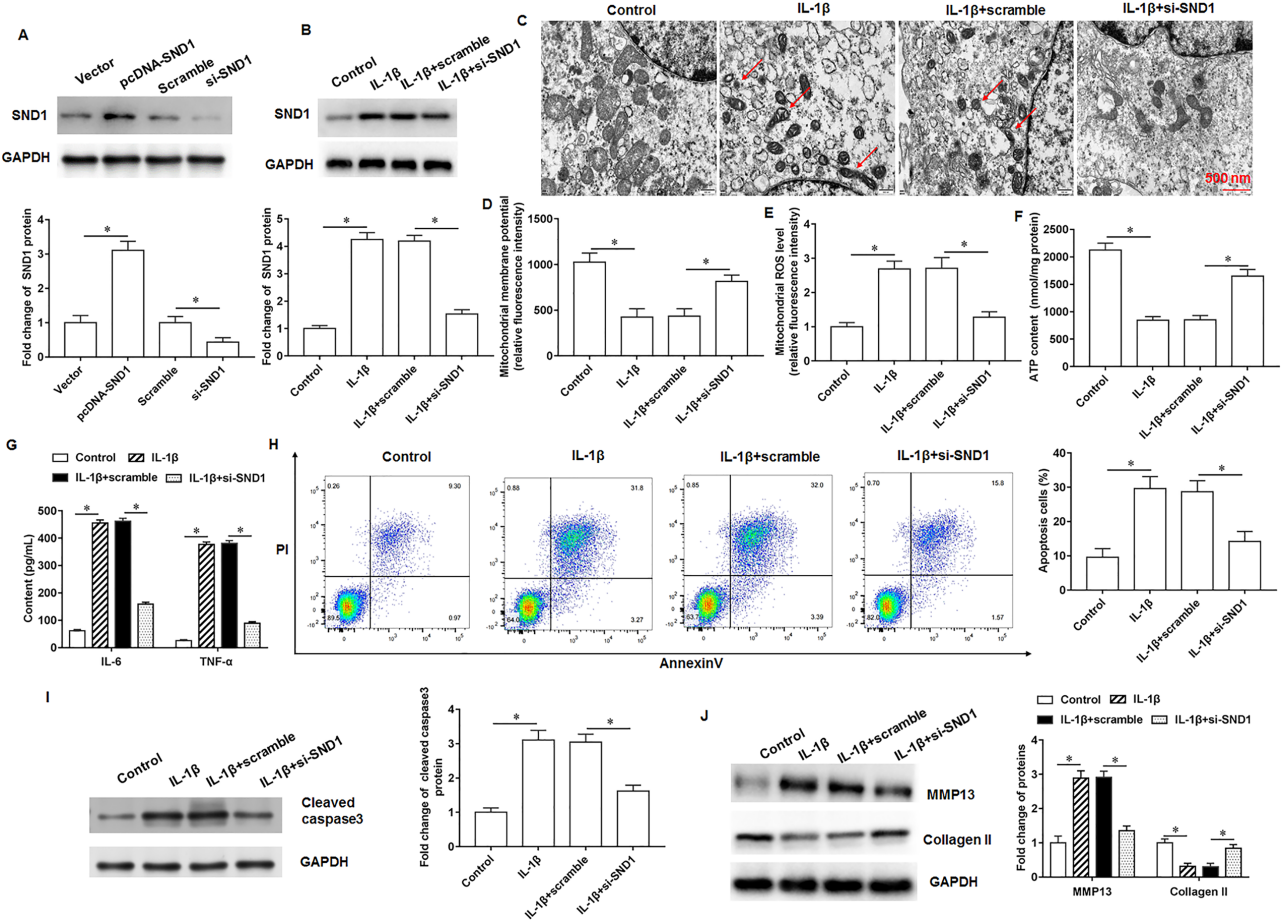
Effects of SND1 on IL-1β-treated chondrocytes. **A**. Transfection efficiency of pcDNA-SND1 or si-SND1 in chondrocytes. **B**. SND1 protein expression. **C**. Representative images of mitochondrial structure under electron microscopic field. **D**. Mitochondrial membrane potential levels. **E**. ROS content detection. **F**. ATP content detection. **G**. Inflammatory factor IL-6 and TNF-α secretion levels. **H**. Cell apoptosis. **I**, **J**. Cleaved caspase3, MMP13 and collagen II protein expression. *N* = 6. *p < 0.01

We transfected pcDNA-SND1 or si-SND1 in chondrocytes, respectively, and the transfection efficiency is shown in Fig. 2A. The SND1 expression was increased in IL-1β-treated chondrocytes, and si-SND1 downregulated SND1 expression after IL-1β treatment (Fig. 2B). Under the electron microscopic field, we found that IL-1β increased chondrocyte intracellular vacuoles, swollen and deformed mitochondria, and si-SND1 decreased the number of chondrocyte intracellular vacuoles, and prevented mitochondrial damage caused by IL-1β (Fig. 2C). Furthermore, mitochondrial membrane potential was decreased (Fig. 2D), ROS content was increased (Fig. 2E), and ATP production was decreased (Fig. 2F) in IL-1β-treated chondrocytes, and si-SND1 reversed the effects of IL-1β. ELISA results showed that IL-1β promoted inflammatory factor secretion, and knockdown of SND1 expression reduced inflammatory factor secretion (Fig. 2G). We observed that IL-1β promoted chondrocyte apoptosis (Fig. 2H), increased cleaved caspase3 (Fig. 2I) and MMP13 (Fig. 2J) protein expression; and reduced collagen II protein expression, and si-SND1 reversed the effect of IL-1β.

### SND1 upregulates PINK1 expression

**Fig. 3:**
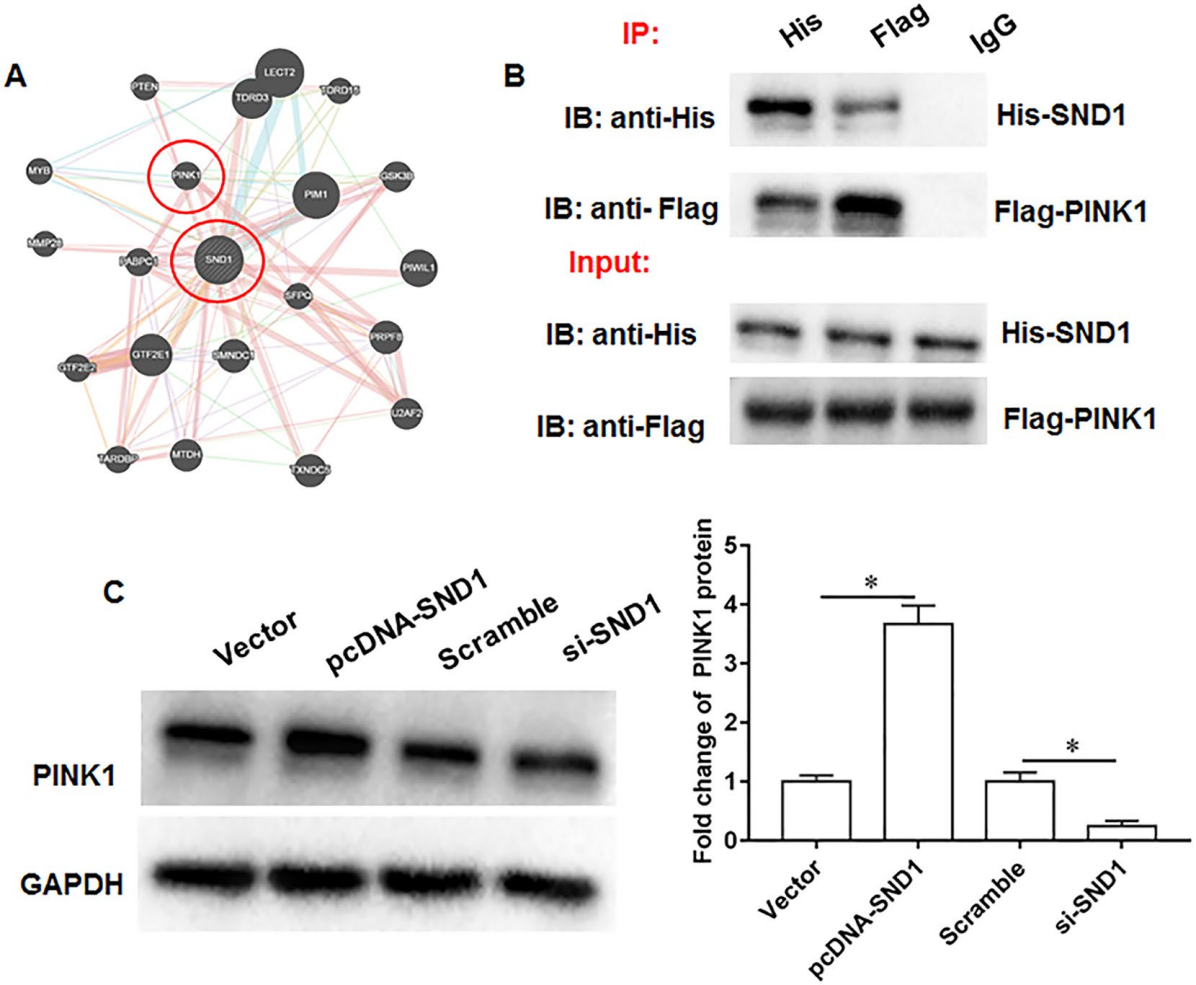
PINK1 is a potential binding protein for SND1. **A**. Online bioinformatics databases were used to predict potential binding proteins of SND1 (https://genemania.org/s; https://thebiogrid.org/). **B**. The binding of SND1 and PINK1 was validated by Co-IP assay. **C.** PINK1 protein expression. *N* = 6. *p < 0.01

Through online bioinformatics analysis, we found that the PINK1 protein may bind to and be regulated by SND1 (https://thebiogrid.org; https://genemania.org/s; Fig. 3A). And co-immunoprecipitation assay verified the binding relationship between SND1 and PINK1 (Fig. 3B). Next, the mouse chondrocytes were transfected with pcDNA-SND1 or si-SND1. And pcDNA-SND1 promoted PINK1 expression and si-SND1 inhibited PINK1 expression (Fig. 3C).

### SND1 promotes mitochondrial damage and matrix degradation in chondrocytes by upregulating PINK1 expression

**Fig. 4:**
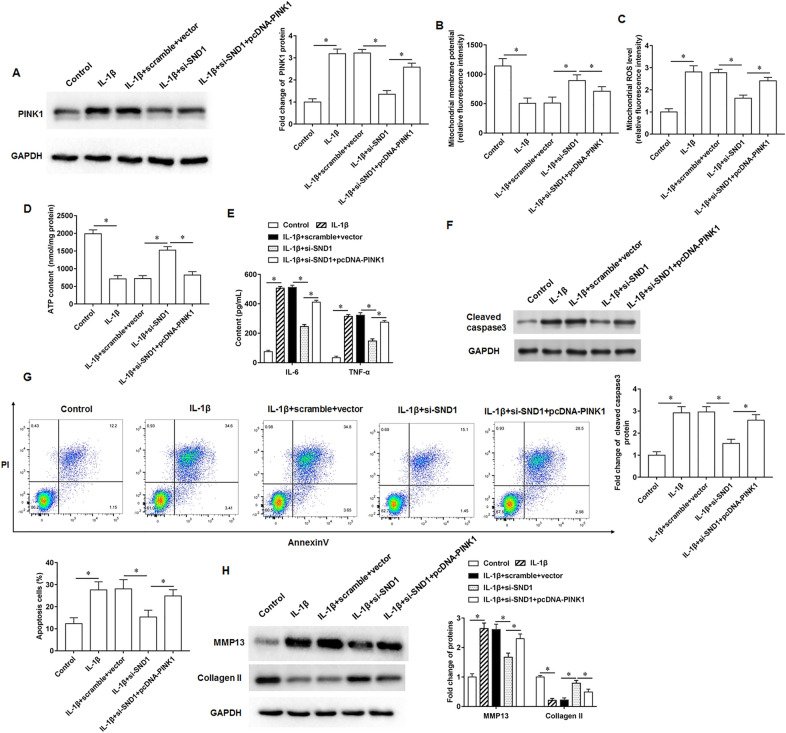
SND1 affects IL-1β-treated chondrocytes through upregulation of PINK1. The IL-1β-treated chondrocytes were transfected with si-SND1 alone or together with pcDNA-PINK1. **A**. PINK1 protein expression.** B**. Mitochondrial membrane potential levels. **C**. ROS content detection.** D**. ATP content detection. **E.** Inflammatory factor IL-6 and TNF-α secretion levels. **F**. Cleaved caspase3 protein expression. **G**. Cell apoptosis. **H**. MMP13 and collagen II protein expression. *N* = 6. *p < 0.01

The IL-1β-treated chondrocytes were transfected with si-SND1 alone or together with pcDNA-PINK1. IL-1β treatment promoted PINK1 protein expression, and si-SND1 inhibited PINK1 protein expression, and pcDNA-PINK1 again promoted PINK1 expression (Fig. 4A). IL-1β treatment decreased mitochondrial membrane potential (Fig. 4B), increased ROS content (Fig. 4C), and decreased ATP generation (Fig. 4D) in chondrocytes,and si-SND1 inhibited the effects of IL-1β, and pcDNA-PINK1 once again reversed the inhibitory effect of si-SND1 on mitochondrial damage. ELISA results showed that IL-1β treatment promoted chondrocyte inflammatory factor secretion, which was reduced after knockdown of SND1 expression and increased again after overexpression of PINK1 (Fig. 4E). We observed that si-SND1 removed the promoting effect of IL-1β on cleaved caspase3 protein expression (Fig. 4F) and chondrocyte apoptosis (Fig. 4G), and pcDNA-PINK1 reversed the changes. Moreover, IL-1β treatment increased MMP13 and decreased collagen II protein expression, and si-SND1 inhibited the effects of IL-1β, and the regulatory effects of si-SND1 on cell behavior were reversed by pcDNA-PINK1 (Fig. 4H).

### PINK1 upregulates BECN1 expression

**Fig. 5:**
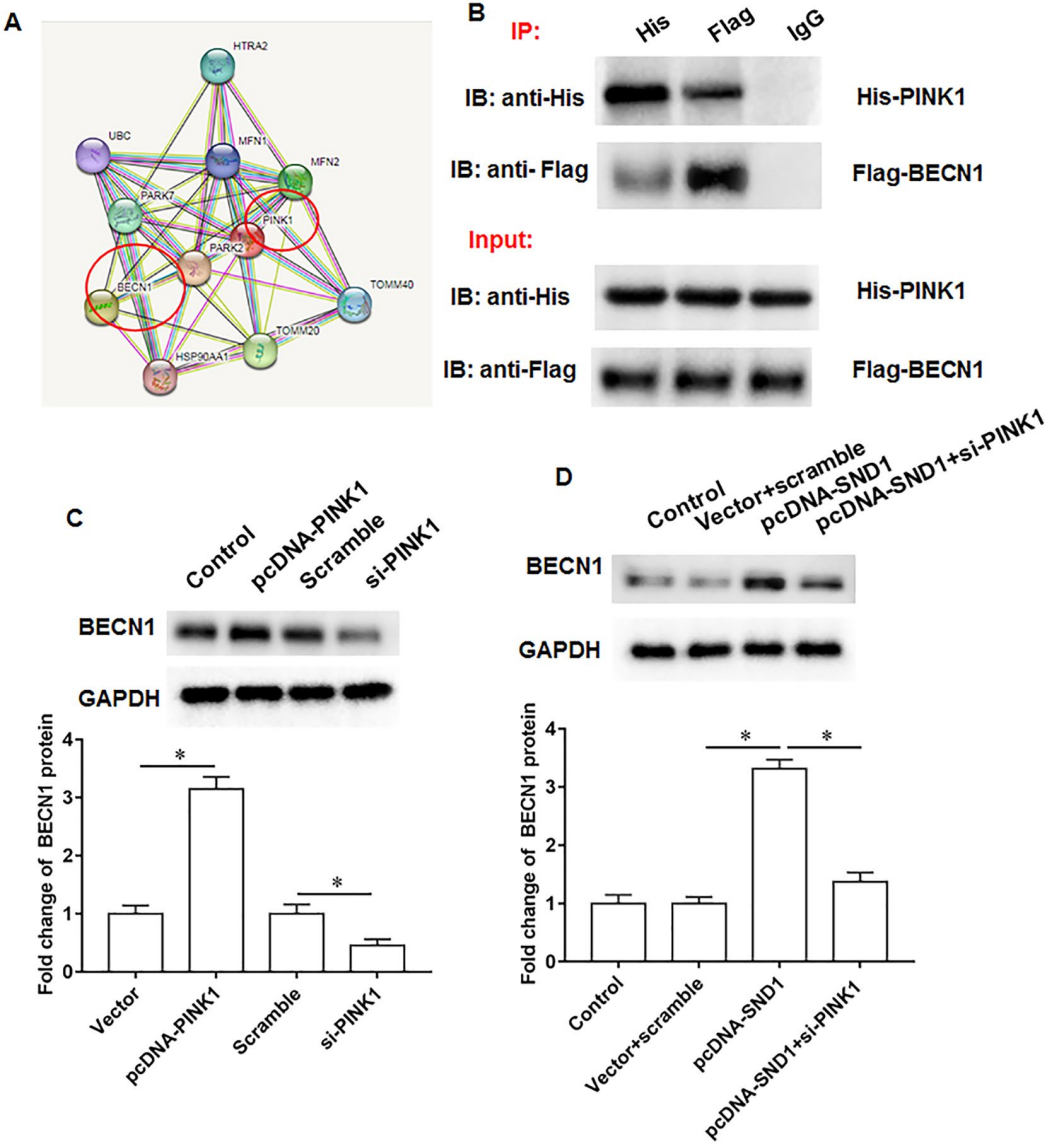
BECN1 is a potential binding protein forPINK1. **A**. Online bioinformatics databases were used to predict potential binding proteins of DRP1 (https://cn.string-db.org/). **B**. The binding of PINK1 and BECN1 was validated by Co-immunoprecipitation assay. **C.** BECN1 protein expression. **D**. BECN1 protein expression. *N* = 6. *p < 0.01

Through online bioinformatics analysis, we found that the BECN1 protein may bind to and be regulated by PINK1 (https://cn.string-db.org/; Fig. 5A). And co-immunoprecipitation assay verified the binding relationship between PINK1 and BECN1 (Fig. 5B). Next, the cells were transfected with pcDNA-PINK1 or si-PINK1. And pcDNA-PINK1 promoted BECN1 expression and si-PINK1 inhibited BECN1 expression (Fig. 5C). Moreover, the cells were transfected with pcDNA-SND1 alone or together with si-PINK1, and SND1 promoted BECN1 expression and si-PINK1 reversed the effect of pcDNA-SND1 (Fig. 5D).

### PINK1 promotes mitochondrial damage and matrix degradation in chondrocytes by upregulating BECN1 expression

**Fig. 6:**
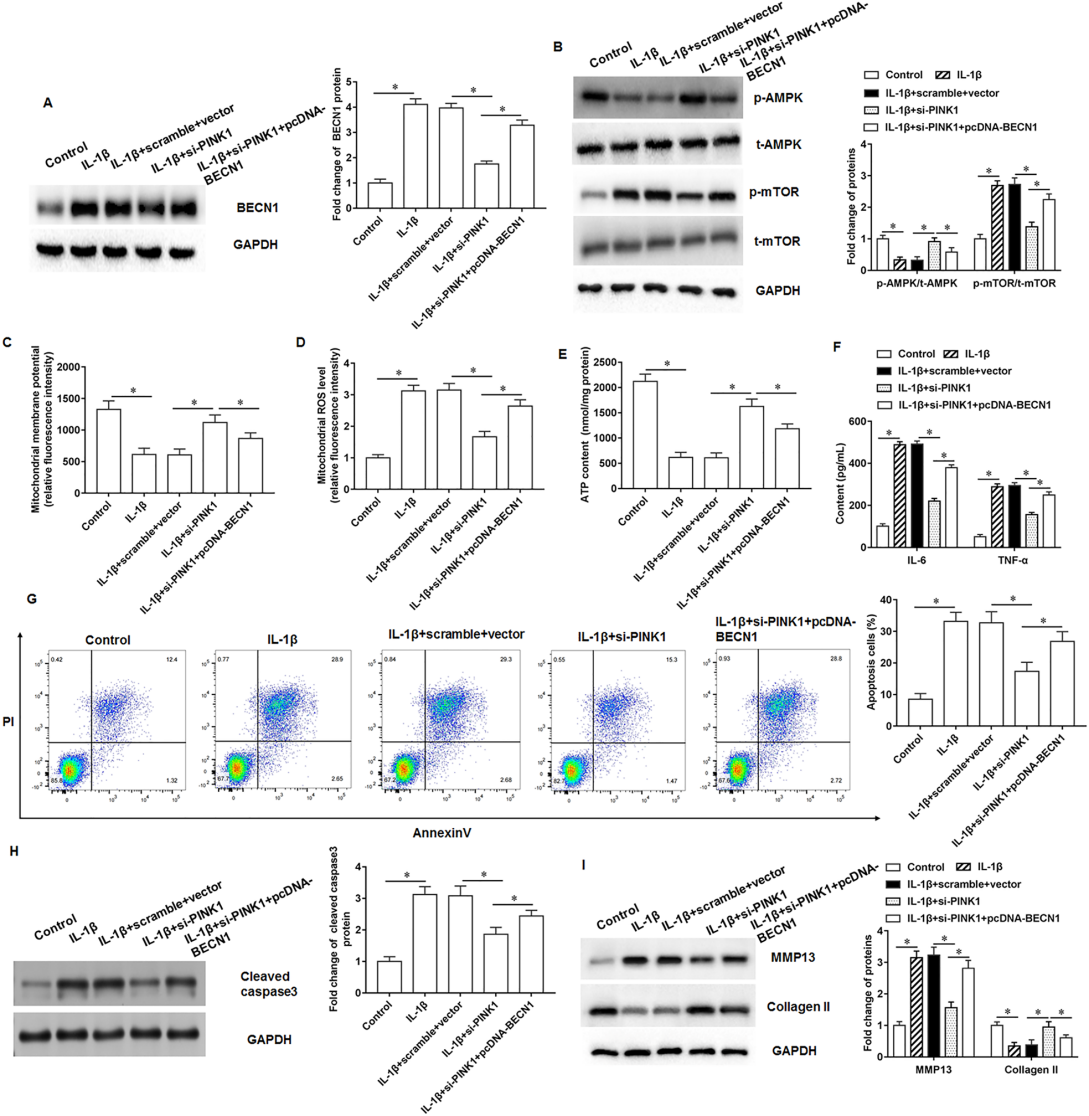
PINK1 affects IL-1β-treated chondrocytes through upregulation of BECN1. The IL-1β-treated chondrocytes were transfected with si-PINK1 alone or together with pcDNA-BECN1. **A**. BECN1 protein expression. **B**. AMPK and mTOR phosphorylation levels. **C**. Mitochondrial membrane potential levels. **D**. ROS content detection. **E**. ATP content detection. **F**. Inflammatory factors IL-6 and TNF-α secretion levels. **G**. Cell apoptosis. **H**, **I**. Cleaved caspase3, MMP13 and collagen II protein expression. *N* = 6. *p < 0.01

The IL-1β-treated chondrocytes were transfected with si-PINK1 alone or together with pcDNA-BECN1. IL-1β treatment promoted BECN1 expression, and si-PINK1 inhibited BECN1 expression, and BECN1 reversed the effect of si-PINK1 (Fig. 6A). Western blotting results suggested that IL-1 treatment decreased the phosphorylation levels of AMPK and mTOR, and knockdown of PINK1 reversed the effects of IL-1, but overexpression of BECN1 increased the phosphorylation levels of AMPK and mTOR (Fig. 6B). Furthermore, IL-1β treatment decreased mitochondrial membrane potential (Fig. 6C), increased ROS content (Fig. 6D), and promoted ATP generation (Fig. 6E) in chondrocytes, and si-PINK1 removed the regulation effects of IL-1β, but mitochondria damage induced by IL-1β was reversed by pcDNA-BECN1. ELISA results showed that IL-1β treatment promoted chondrocyte inflammatory factor secretion, which was reduced after knockdown of PINK1 expression and increased again after overexpression of BECN1 (Fig. 6F). We observed that si-PINK1 removed the promoting effect of IL-1β on chondrocyte apoptosis, and BECN1 overexpression had the opposite effect to si-PINK1 (Fig. 6G). Moreover, IL-1β treatment increased cleaved caspase3 and MMP13 protein expression, and decreased collagen II protein expression, and si-PINK1 reversed the effect of IL-1β, and pcDNA-BECN1 again increased cleaved caspase3 (Fig. 6H) and MMP13, and decreased collagen II protein expression (Fig. 6I).

### Knockdown of SND1 expression alleviates osteoarthritis progression in rats

**Fig. 7:**
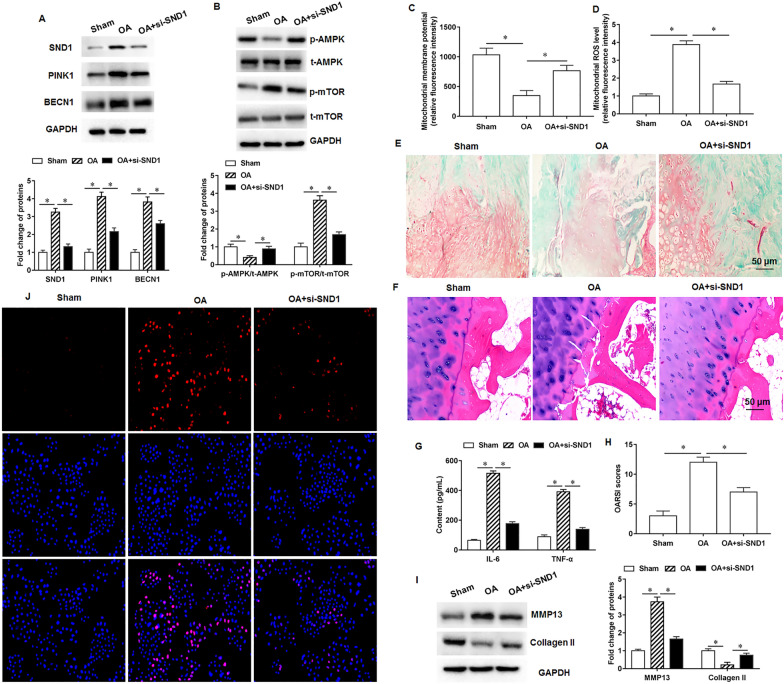
Effects of knockdown of SND1 expression on osteoarthritis progression in rats. Eighteen SPF male SD rats (8–10 weeks old, weighing 280–300 g) were randomly divided into three groups with 6 rats in each group, namely sham group, osteoarthritis model group and si-SND1 group. **A**. The SND1, PINK1 and BECN1 protein expression. **B**. AMPK and mTOR phosphorylation levels. **C**. Mitochondrial membrane potential levels. **D**. ROS content detection. **E**. The safranin O-fast green staining. **F**. HE staining. **G**. Inflammatory factors IL-6 and TNF-α secretion levels. **H**. Cartilage tissue score in rats. **I**. MMP13 and collagen II protein expression. **J**. Cell apoptosis. *N* = 6. *p < 0.01

In vivo results showed that SND1 silencing inhibited SND1, PINK1 and BECN1 proteins expression in rat knee cartilage tissues compared with the osteoarthritis rats (Fig. 7A). Furthermore, SND1 silencing reduced AMPK and mTOR phosphorylation levels in knee joint cartilage tissues of osteoarthritis rat (Fig. 7B). We observed increased mitochondrial membrane potential (Fig. 7C) and decreased ROS content (Fig. 7D) in cartilage tissues rats with SND1 silencing. Furthermore, the safranin O-fast green staining (Fig. 7E) and HE staining (Fig. 7F) results suggested that SND1 silencing was beneficial to the repair of damaged cartilage tissue. ELISA results indicated that inflammatory factor secretion was reduced in the knee joint cartilage tissues of osteoarthritis rats with SND1 silencing (Fig. 7G). Compared with the rats in the model group, the cartilage tissue scores of osteoarthritis rats decreased under the treatment of si-SND1, suggesting that the cartilage damage of rats was alleviated (Fig. 7H). Furthermore, knockdown of SND1 expression inhibited MMP13 and promoted collagen II protein expression in osteoarthritis rat knee joint cartilage tissues (Fig. 7I). TUNEL staining results indicated that osteoarthritis rat knee chondrocyte apoptosis increased, and cell apoptosis decreased after knockdown of SND1 (Fig. 7J).

## Discussion

Osteoarthritis is a chronic degenerative disease, and numerous studies have shown that the progression of osteoarthritis is associated with mitochondrial dysfunction. Numerous experiments have observed that osteoarthritic articular chondrocytes exhibit impaired mitochondrial electron transport chain, decreased mitochondrial membrane potential, increased levels of ROS, and swollen and deformed mitochondria, which can cause chondrocyte apoptosis [19, 20]. Additionally, osteoarthritis is also associated with reduced expression of manganese superoxide dismutase and oxidative damage to mitochondrial DNA in superficial chondrocytes of articular cartilage. Taken together, these findings suggest that mitochondrial dysfunction and oxidative damage play a key role in the pathogenesis of osteoarthritis.

PTEN mediated putative kinase protein 1 (PINK1) is a protein kinase present in mitochondria mainly with serine or threonine proteins as substrates and is encoded by the PINK1 gene. PINK1 protein is widely expressed, is composed of 581 amino acid residues, and possesses a highly conserved domain. PINK1 contains an NH2 terminal mitochondrial targeting sequence, and many models suggest that PINK1, which accumulates on the outer mitochondrial membrane after cellular stress to recruit parkin and clear mitochondria by mitophagy, is a mitophagy related protein kinase [21, 22]. PINK1 can enter mitochondria to regulate the degradation of mitochondrial respiratory chain subunits, with an important role in mitochondrial function. In osteoarthritis progression, a study has reported that activation of the PINK1/parkin pathway accelerates mitophagy, contributing to the maintenance of mitochondrial homeostasis and the alleviation of chondrocyte injury [23]. Moreover, Wang et al. provided evidence that metformin inhibitsIL-1β-induced oxidative stress and inflammation in chondrocytes by enhancing the SIRT3/PINK1/parkin signaling pathway [24]. In this study, we found that PINK1 expression was increased in IL-1β-stimulated mouse chondrocytes, and overexpression of PINK1 enhanced IL-1β-induced mitophagy defects, decreased ATP levels, and oxidative damage. This may be contrary to the findings of other scholars. However, our findings have also received relevant support. Shin et al. showed that the expression of PINK1 and parkin proteins increased in osteoarthritis, and PINK1 mediated mitophagy in chondrocytes led to cartilage degeneration in osteoarthritis [25]. We consider that the reason for the opposite of the research results is that mitophagy is an extremely complex physiological process, and its essence is a selective autophagy, which plays a role in stabilizing the intracellular environment by maintaining the balance of the quality and quantity of mitochondria, but under stress conditions, both insufficient autophagy and excessive autophagy will damage cells.

Normally, damaged cells clear cell debris by mitophagy to restore homeostasis, but excessive mitophagy activation and mitochondrial dysfunction lead to apoptosis. A study has shown the investigation that over activation of PINK1/parkin pathway is an important cause of OPD induced mitophagy and mitochondrial damage in cardiomyocytes [26]. Moreover, one study found that inhibition of mitophagy by mdivi-1 attenuates the PINK1/parkin pathway activation by doxorubicin and protects mitochondrial biogenesis, resulting in the alleviation of doxorubicin induced mitochondrial superoxide generation and mitochondrial dysfunction [27]. Interestingly, activation of the PINK1/parkin pathway is currently likewise reported to lead to apoptosis of osteoarthritic chondrocytes. The investigators found that PINK1 protein expression was increased during osteoarthritis progression and that PINK1 mediated mitosis accelerated the death of monosodium iodoacetate stimulated chondrocytes, whereas loss of PINK1 expression attenuated chondrocyte injury and pain behaviors in rats with osteoarthritis [25]. Now, we showed that PINK1 protein expression was increased in osteoarthritic chondrocytes and caused decreased mitochondrial membrane potential, increased ROS generation, decreased ATP content, and increased apoptosis, which may be closely related to overactivated mitophagy.

AMP-activated protein kinase (AMPK) is a serine/threonine protein kinase that coordinates metabolism and energy requirements and is also a central player in glucose and lipid metabolism. One study found that in H_2_O_2_ stimulated rat chondrocytes, globular adiponectin reduced chondrocyte apoptosis by increasing the level of AMPK phosphorylation and decreasing the level of mTOR phosphorylation, which was mediated through AMPK/mTOR signaling pathway activation associated mitophagy [28]. Moreover, some scholars reported that puerarin increased mitochondrial biogenesis and alleviated mitochondrial dysfunction in chondrocytes of osteoarthritis rats, but AMPK inhibitor was able to abolish the effect of puerarin, suggesting that activation of AMPK pathway is of great significance for mitochondrial repair in osteoarthritis chondrocytes [29]. In this study, we found that the AMPK pathway was blocked and mitochondrial function was impaired in IL-1β-stimulated chondrocytes, and the phosphorylation level of AMPK was increased and mitochondrial function was restored when SND1 or PINK1 expression was knocked down, which was consistent with the findings of other scholars.

In this study, we first found that SND1 was upregulated in chondrocytes of patients with osteoarthritis. Secondly, in IL-1β-stimulated mouse chondrocytes, SND1 expression was increased, and SND1 promoted PINK1 expression, and PINK1 promoted BECN1 expression, accelerating mitochondrial damage and matrix degradation in mouse chondrocytes. In addition, the protein expressions of SND1, PINK1 and BECN1 were increased in the rat OA model, and SND1 silencing alleviated mitochondrial damage and cartilage degradation in osteoarthritis rats, indicating that the mechanism in the in vivo model is the same as that in the in vitro inflammatory model. However, there are still some deficiencies in this study. In all the results, although we detected the transfection efficiency of siRNA, we only used one siRNA for research, and the types of siRNA used were not enough. In addition, it may be related to the tissue section making method or staining procedure. The results of safranin fast green staining of rat cartilage tissue obtained are not ideal, and do not clearly show the morphology of articular cartilage. In the later research, we will seek more professional personnel to guide our experimental operation to improve the quality of the article.

## Data Availability

All the data obtained in the current study were available from the corresponding authors on reasonable request.
